# Effects of elevated temperature on the corrosion resistance of silver–cobalt oxide–titanium dioxide (Ag/Co_3_O_4_/TiO_2_) nanocomposites coating on AISI 1020

**DOI:** 10.1038/s41598-021-90272-w

**Published:** 2021-05-25

**Authors:** Mohammed Ibrahim, Joseph B. Agboola, Saka A. Abdulkareem, Oyewole Adedipe, Jimoh O. Tijani

**Affiliations:** 1 Department of Mechanical Engineering, Federal University of Technology, Minna, Nigeria; 2 Metallurgical and Materials Engineering Department, University of Lagos, Lagos, Nigeria; 3 Department of Chemical Engineering, Federal University of Technology, Minna, Nigeria; 4 Department of Chemistry, Federal University of Technology, Minna, Nigeria

**Keywords:** Materials science, Nanoscience and technology

## Abstract

The effects of temperature on corrosion resistance of Silver–Cobalt oxide and Titanium Dioxide (Ag/Co_3_O_4_/TiO_2_) nanocomposite coated AISI 1020 in a high-temperature environment was investigated. The Ag, Co_3_O_4_ and TiO_2_ nanoparticles were individually produced by mixing the salt precursors with extract of *Piptadeniastrum Africana* leaf under the optimized synthesis conditions. The nanocomposite was produced by mixing Ag, Co_3_O_4_ and TiO_2_ nanoparticles (NPs) in equal proportions to constitute 75 wt% of the composite. 10 wt% epoxy resin and its hardener in the ratio (1:1) were added to serve as the binder, while 15 wt% of CNT was introduced to serve as support. The produced Ag/Co_3_O_4_/TiO_2_ nanocomposite was coated on the surface of mild steel (AISI 1020) by the dipping method. The coated samples were heated in a muffle furnace to 35, 100, 200, and 300 °C. Microstructural evolution of the coatings was investigated using X-ray diffraction, scanning electron microscopy and energy dispersive spectrometer. The corrosion resistance of the coated and heated and un-heated steel samples was determined using the potentiodynamic polarization method. The results show that Ag/Co_3_O_4_/TiO_2_ nanocomposite coated sample cured at 100 °C exhibited the highest corrosion resistance of 195.12 Ω.

## Introduction

Mild steel accounts for over 98% of the construction materials due to its remarkable mechanical properties such as good strength, toughness, ductility, formability, weldability and availability^1^. However, the problem with mild steel is corrosion particularly in environment of high temperature^2^. Several structural failures have occurred due to corrosion and have resulted to loss of human lives and additional costs on repairs or reconstruction of such infrastructures^3^. In developed countries like United States of America (USA), it is estimated that the cumulative costs resulting from corrosion annually is about 300 Million USD, while in developing countries like Nigeria it is assumed to be about 10 Million USD^4,5^. There are several methods of corrosion control which includes; modification of the micro structures, use of corrosion inhibitors, cathodic protection and coating methods. According to^6^, among these methods, the coating method is considered one of the most effective methods because it provides complete shield of the substrate against the corrosion medium. The development of nanotechnology brought about coatings that performed excellently in terms of corrosion protection of steel in corrosive mediums. Several metals and non-metals, ceramics and polymer materials have been applied as coating on mild steel to improve their surface engineering properties such as hardness, wear, fatigue and corrosion resistant. Kavitha et al.^7^ reported that conventional organic coatings such as epoxy coatings fail at high temperature resulting to cracks, delamination, blisters and pore formation which affects the corrosion protection properties. Mathiazhagan et al.^8^ studied electro deposition of Ni–Cr_2_O_3_ nanocomposites and showed that the application of these nanocomposites on mild steel surface improved the tribological and mechanical properties of the steel. Seidu and Kutelu^9^ produced a ternary Zn–Cr_2_O_3_–SiO_2_ nanocomposite coating on mild steel using electrolytic chloride bath solution and reported an improvement in tribological behaviour and better thermal stability of the nanocomposite coating. Abd et al.^10^ investigated the corrosion resistance of Zn–Co–TiO_2_ nanocomposite and reported an improved interfacial interaction between the nanoparticles which lead to improved corrosion resistance. Despite the wonderful performance of metal and metal oxide nanoparticles, certain limitations such as particle agglomeration that often creates voids and coating cracks through which corrosion is initiated and propagated have been identified. Secondly, premature failure due to high temperature environment and lack of adequate adherence to substrate have been reported. Silver and cobalt oxide nanoparticles have been known for their excellent corrosion resistance properties while titanium dioxide nanoparticles have good heat resistance as well as corrosion resistance properties. The use of these materials as anti-corrosion coating for mild steel under high temperature condition is scarce in literature and therefore is the focus of this research work”.

## Materials and methods

### Materials

The AISI 1020 steel rod used in this study was obtained from scrap market in Minna, Niger State, Nigeria. Silver nitrate (AgNO_3_) at 99.8% purity, cobalt(II) nitrate, Co(NO_3_)_2_·6H_2_O, (97%), titanium(IV) isopropoxide (97%), carbon nanotubes (CNTs) (95%), concentrated hydrochloric acid (HCl) (90%), sodium hydroxide (NaOH) (90%) distilled water (95%), acetone (97%), aluminium chloride (99.5%), sodium acetate, sodium carbonate (Na_2_CO_3_), (95%), 10% Folin–Ciocalteau’s Reagent (95%) were obtained from sigma Aldrich, China and Kamel, England. Emery papers of 220, 400, 600 and 800 grits, magnetic stirrer, freeze drier, ultra-sonic bath, high speed refrigerated centrifuge, high resolution SEM.

### Methods

#### Sample preparation

AISI 1020 mild steel rod of 10 mm diameter was used for this work. Ten (10) samples were cut according to ASTM standard for corrosion test. Eight (8) of the samples were heated treated to a temperature of 850 °C to relieve the samples of machining stress, followed by grinding with emery papers of 220, 400, 600 and 800 grits, washed with distilled water and degreased in acetone. Two (2) of the samples were designated as control.

#### Synthesis of Ag, Co_3_O_4_ and TiO_2_ nanoparticles

**Figure 1:**
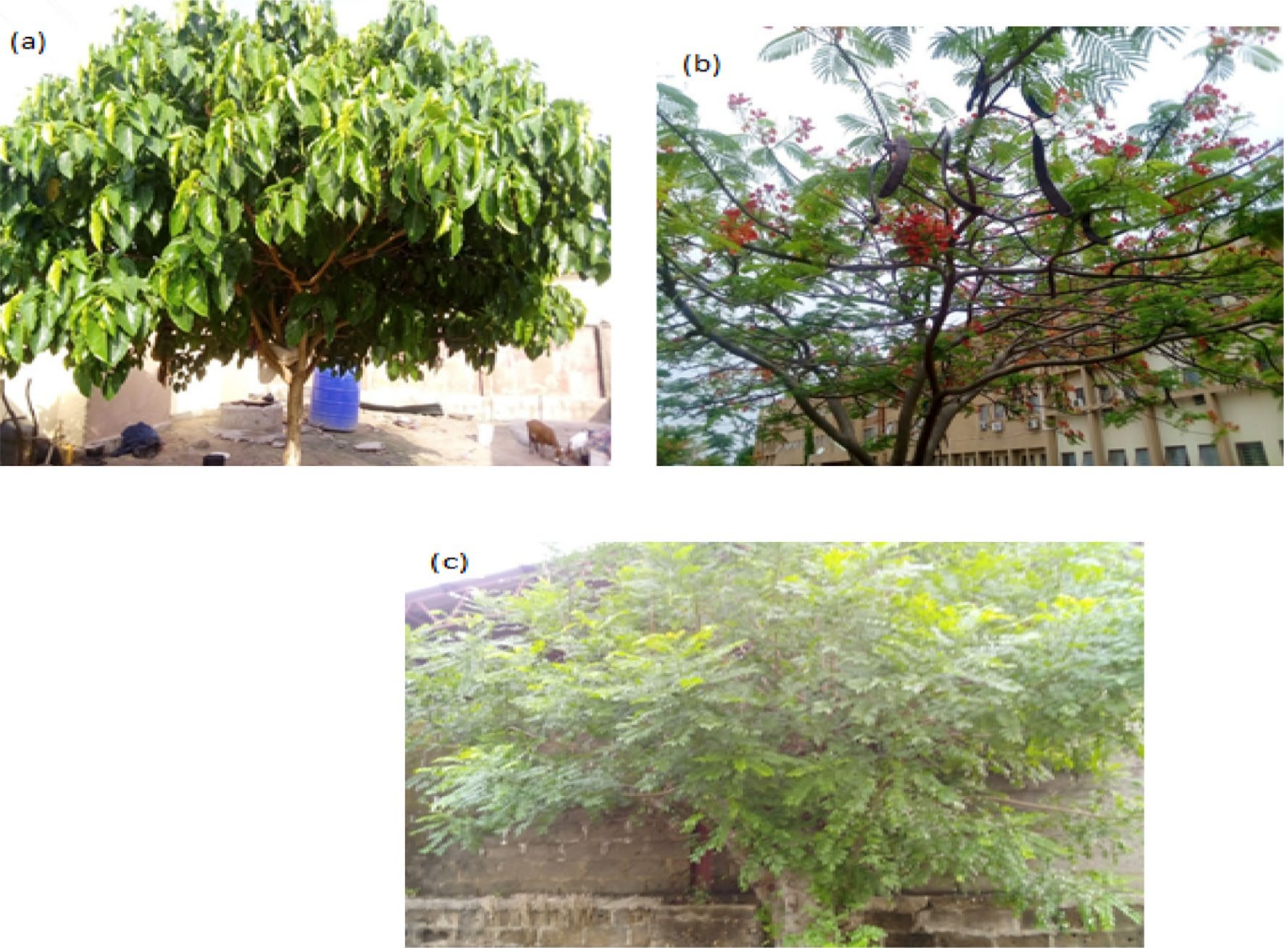
(**a**) *Ficus thongili*, (**b**) *Piptadeniastrum africana*, (**c**) *Ximenia americana*.

Fresh leaves of three representative plant samples of *Ficusthongili, Ximenia americana and Piptadeniastrum africana* were collected from Bosso, Kpakungu and Gidan-Kwano areas respectively in Minna, Niger state Nigeria. The leaves were thoroughly washed with running tap water and then with distilled water in order to remove dust and other contaminants and further dried under shed at room temperature for a period of seven (7) days. The dried leaves were pounded vigorously into homogeneous powder using mortar and pestle. The obtained powder was then kept under dry condition for feature use. Figure 1 show the three representative plants from which the leave samples were collected. All the relevant institutional, national, and international guidelines regarding plant usage were strictly observed.

*Piptadeniastrum* Africana leaf extract was utilized for the synthesis of the nanoparticles. Silver nanoparticles were synthesized by a mixture of 1 ml of the extract and 10 ml of aqueous AgNO_3_ under a pH of 8 at a temperature of 35 °C for a reaction time of 60 min., Cobalt Oxide nanoparticles were synthesized by a mixture of 10 ml of the extract and 50 ml of 1 × 10^−3^ M aqueous Co (NO_3_)_2_.6H_2_O solution at an incubation temperature of 40 °C for 60 min. The TiO_2_ NPs were synthesized by adding 20 ml of distilled water to 20 ml of aqueous leaf extract in an Erlenmeyer flask under continuous stirring condition using magnetic stirrer and then 10 ml of aqueous titanium tetraisopropoxide solution was added drop wise. The mixture was kept under continuous stirring at room temperature for two (2) hours. The formed nanoparticles were acquired by washing severally with distilled water. The colloidal nanoparticles were oven dried at 100 °C for 2 h. The dried powder was then calcined at 450 °C for 2 h in a muffle furnace.

#### Synthesis of carbon nanotubes (CNT)

1 g of the calcined Fe–Ni alloy catalyst was measured in ceramic quartz boot using weighing balance, the weight of the catalyst and the quartz boot was recorded, the quartz boot was inserted in the furnace tube and was adjusted to the centre of the reaction chamber of the CVD equipment The CVD equipment was programmed to heat at 10 °C per minute to the temperature of 750 °C. The Argon which served as the carrier gas for the carbon source (ethylene) was initially set to flow at 30 ml/min, to purge the furnace as the temperature rises to 750 °C. This was done to flush all impurity gases that could affect the catalytic activities in the tube. As the temperature reached 750 °C, the flow rate of the Argon was increased to 230 ml/min and the carbon source (ethylene) which was initially programmed to flow at 200 ml/min was introduced. According to the CVD inputted program, the furnace was set to hold at 750 °C for 60 min. After the process, the CVD was allowed to cool to ambient temperature, the quartz boot with the produced CNTs were collected and weigh to evaluate the percentage yield of the CNTs. The CNTs appeared in form of clump, which was gently ground to particles using ceramic mortar and pestle.

#### Preparation of silver/cobalt oxide/titanium dioxide (Ag/Co_3_O_4_/TiO_2_) nanocomposite using CNT as support

Ag/Co_3_O_4_/TiO_2_ nanocomposite was produced by mixing Ag, Co_3_O_4_ and TiO_2_ NPs in equal proportion which add up to 75 wt% of the composite. 10 wt% of epoxy resin and its hardener in the ratio (1:1) were added to serve as the binder, while 15 wt% of CNT was introduced to serve as support. The mixture was stirred at a speed of 1200 rpm for 15 min using magnetic stirrer and sonicated in acetone for another 15 min using ultrasonic bath.5% epoxy resin was then added and the mixture was further stirred and sonicated for further 15 min. Finally, 5% epoxy hardener was added and the resulting mixture was stirred for another 10 min.

#### Characterization of mild steel and seawater samples

The sample of the obtained mild steel was characterized by X-ray fluorescence (XRF) for chemical composition, while the sample of seawater obtained from Lagos lagoon was characterized by chromatographic analysis. In other to view the morphology of the mild steel samples before and after corrosion, high resolution scanning electron microscopy (HRSEM) was used for the analysis.

### Coating of the steel substrates

The samples were coated using dip coating techniques and then dried at various temperatures of 35, 100, 200 and 300 °C.

### Potentio-dynamic polarization test

The potentio-dynamic polarization measurements were carried out with the aid of a computer-controlled Potentiostat/galvanostat EGG 273A coupled to a frequency response analyzer in a conventional three-electrode cell system under static laboratory conditions. The corrosion cell was made up of reference electrode which was a saturated calomel electrode (SCE), the counter electrode was a platinum grid and the working electrode (WE) was the nano coated mild steel samples. The potential (E) of the working electrode were maintained within the range of ± 2.5 V at a scanning rate of 5 mV s^−1^. The working electrode were immersed in the test solution for15 min until a steady state open circuit potential (OCP) were established and the measurements were carried out within the potential range. The experiment was repeated for two more times and average readings were recorded. All electrochemical tests were performed at room temperature. The corrosion rate was calculated using ASTM relation shown in Eq. (1)^11^.

1 $$Corrosion Rate (CR)Mpy=\frac{0.129 \times M \times Icorr}{D}$$

where *CR* is the Corrosion rate Mpy. *M* is the molecular weight of the steel sample = 55.847 g/mol. *I*_*corr*_ is the corrosion current density (I A m^−2^). *D* is the density of the steel = 7.874 kg/m.

The polarization resistance was obtained from Stean Gary equation as shown in Eq. (2). (Shetty and Shetty 2016).

2 $$Rp=\frac{\beta a\beta c}{2.303\left(\beta a+\beta c\right)Icorr}$$

where β_a_ and β_c_ (V dec^−1^) are the Tafel constants obtained from the slopes of anodic and cathodic reaction curves respectively.

## Results and discussion

### Chemical composition

The chemical composition of the steel is presented in Table 1.

**Table 1:** Chemical composition of the As-received AISI 1020.

Element	C	Si	S	P	Mn	Ni	Cr	Mo	V
% composition	0.23	0.038	0.025	0.035	0.43	0.011	0.012	0.012	0.002

Element	Cu	W	As	Sn	Co	Al	Zn	Fe	
% composition	0.004	0.001	0.03	0.02	0,02	0.01	0.03	99.09	

The composition of the metallic substrate shown in Table1 with 0.23 wt% C, 0.43 wt% Mn, 0.035 wt% P, 0.025 wt% S and 99.09 wt% Fe, according to the American Iron and Steel Institute (AISI), corresponds to the composition of AISI 1020 mild steel.

### Characterization of Ag/Co_3_O_4_/TiO_2_ nanocomposite

#### SEM micrographs of Ag, Co_3_O_4_, TiO_2_ nanoparticles and Ag/Co_3_O_4_/TiO_2_ nanoparticles

**Figure 2:**
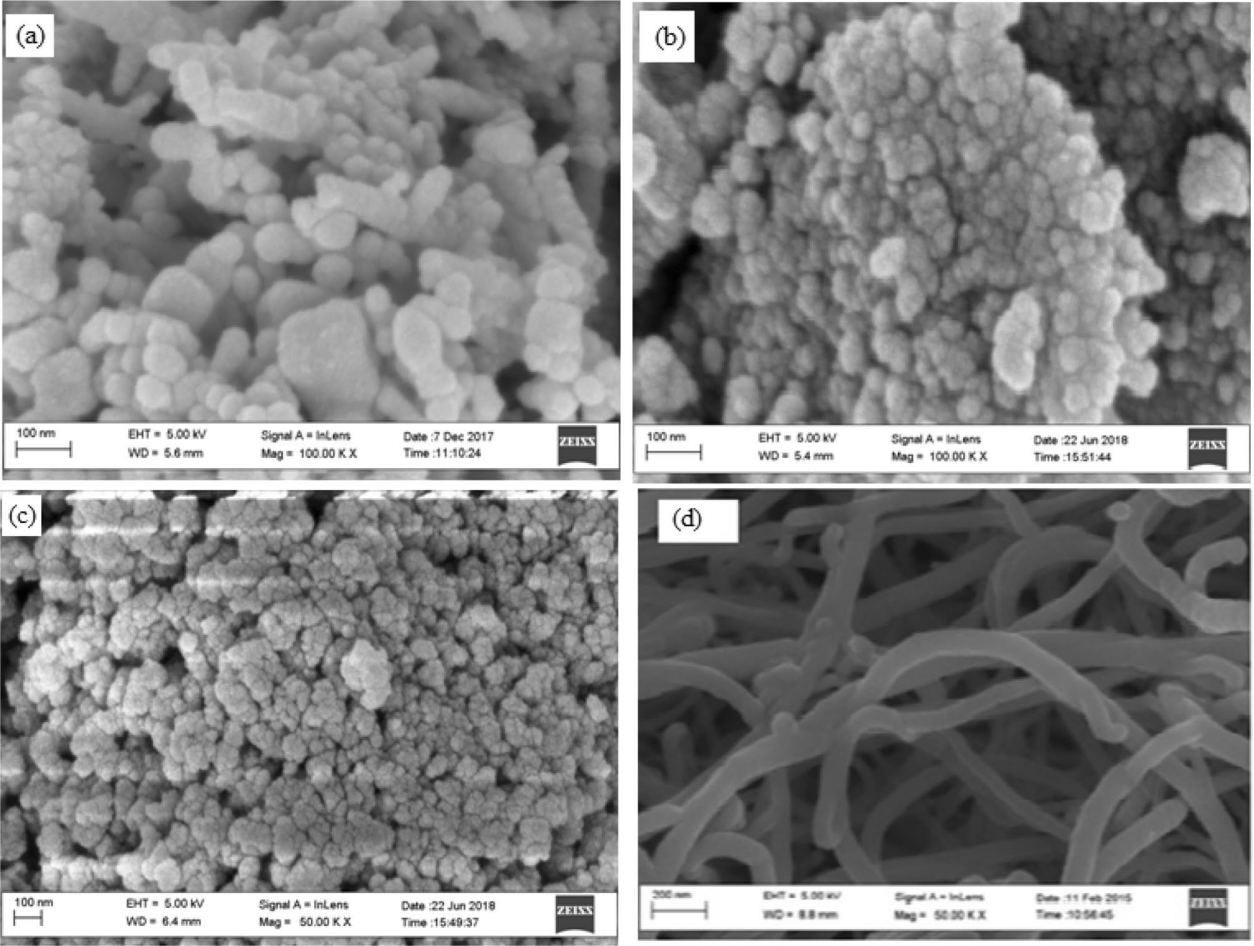
SEM micrographs of (**a**) silver NPs, (**b**) cobalt oxide NPs, (**c**) titanium dioxide NPs and (**d**) purified carbon nanotubes CNTs.

**Figure 3:**
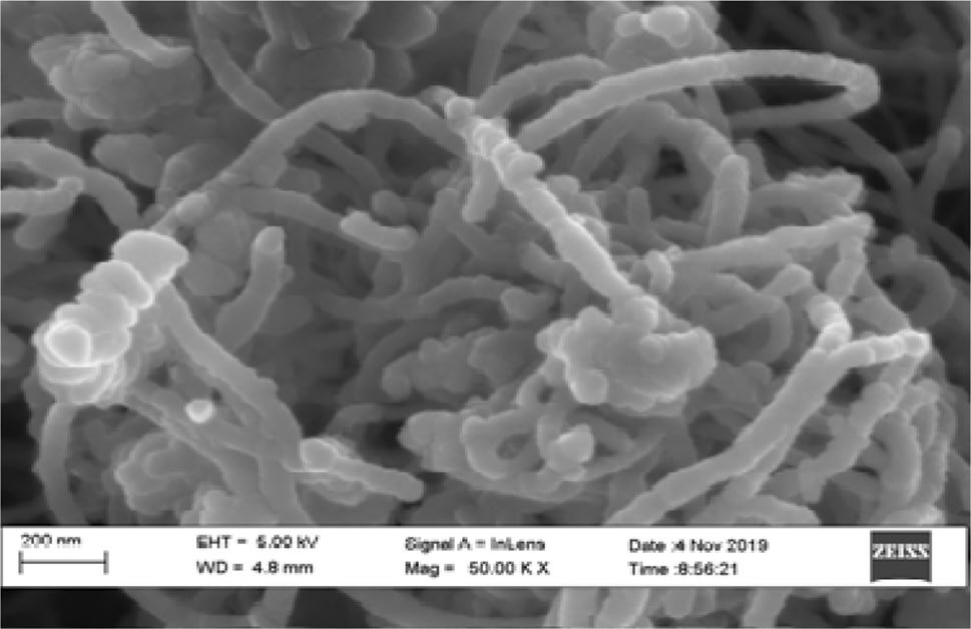
SEM micrographs of Ag/CO_3_O_4_/TiO_2_ nanocomposite.

The SEM micrographs of silver, Cobalt Oxide Titanium dioxide nanoparticle and Purified Carbon nanotubes CNTs are shown in Fig. 2(a)–(d) respectively, while that of the composite is shown in Fig. 3. It can be observed from Fig. 3 that other elements in the composite are embedded in the carbon nanotubes as indicated by the white and dark spots. This is attributed to the large surface area of the CNT that accommodates other nanoparticles and providing reinforcement and better performance of the composite.

#### XRD characterization of Ag/Co/TiO_2_ nanocomposite

**Figure 4:**
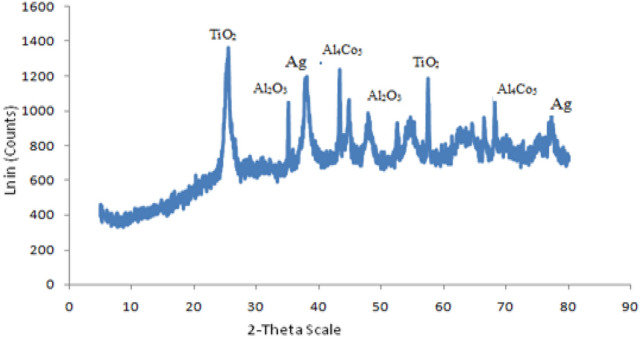
XRD pattern of Ag/Co_3_O_4_/TiO_2_ nanocomposite.

The XRD pattern of Ag/Co_3_O_4_/TiO_2_ nanocomposite is shown in Fig. 4.

The elemental composition of the composite was detected by XRD analysis as shown in Fig. 4. TiO_2_ was detected at 25.4° corresponding to (101) and at 57.3° corresponding to (211) planes in hexagonal packed structure. Ag was detected at 38.2° corresponding to (101) and 78.6° corresponding to (301) planes in fcc structure. Al_2_O_3_ was detected at 35.1° while cobalt existed in form of AlCo_3_ at 41.0° and 68.3°. The presence of aluminium in the form of Al_2_O_3_ and AlCo_3_ were attributed to instrumental impurities. This shows that the composite consisted of the three elements expected to form the composite.

### Electrochemical results

**Figure 5:**
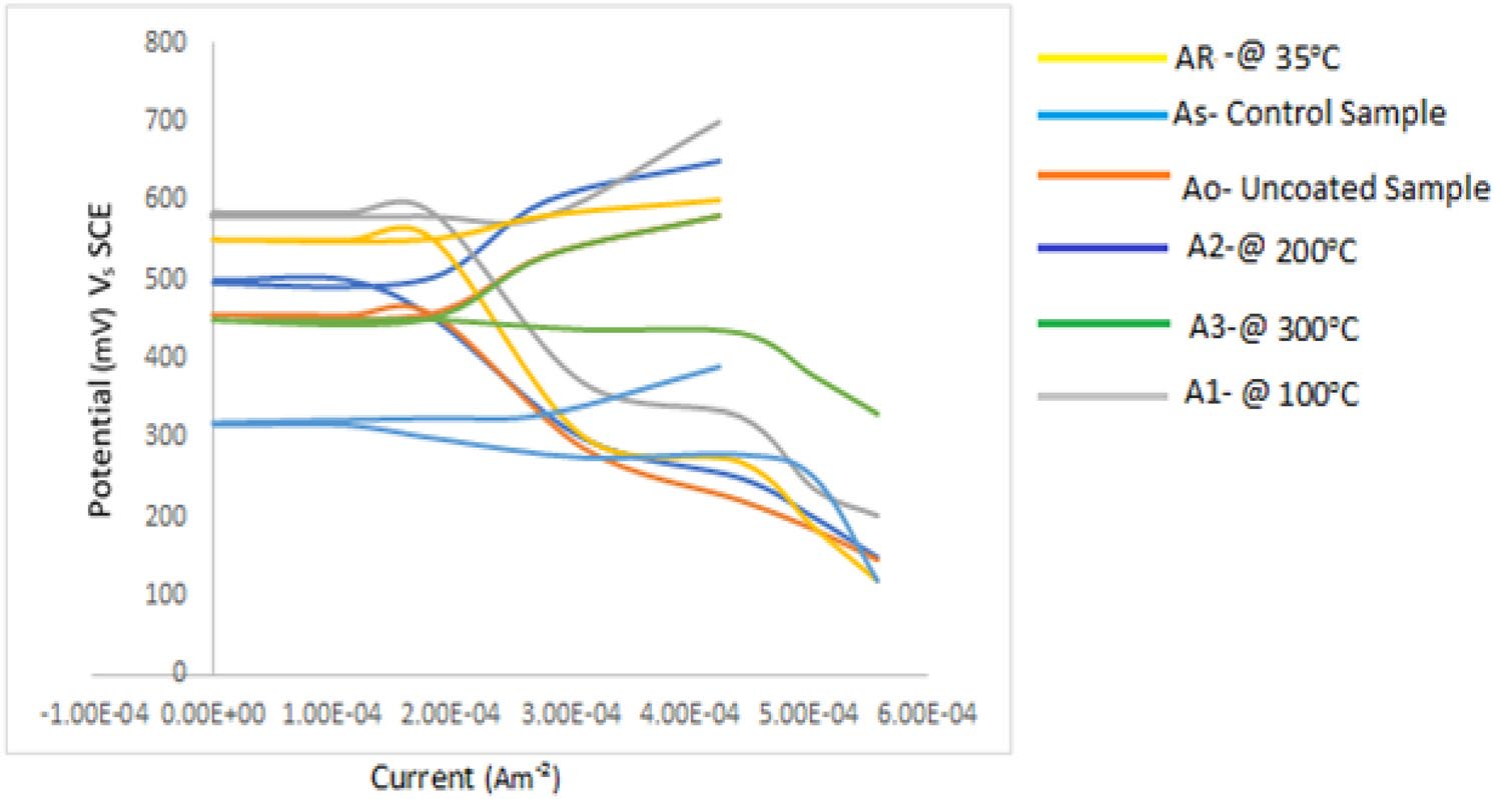
Potentiodynamic curves for Ag/Co_3_O_4_/TiO_2_ nano composite coated AISI 1020 under various temperatures in seawater.

Figure 5 shows the potentiodynamic polarization curves of the as-received and Ag/Co_3_O_4_/TiO_2_ coated mild steel heated at various temperatures in 1.0 M HCl. The corrosion properties estimated from the curves are presented in Table 2. Generally, a high corrosion potential and a low corrosion current density indicates a higher resistance of the material to dissolution in the given electrolyte.

**Table 2:** Corrosion data of Ag/Co_3_O_4_/TiO_2_ nano composite coated AISI 1020 under various temperatures in seawater.

Sample ID	I_corr_ (mA m^−2^)	E_corr_ (mV)	β_a_ (V dec^−1^)	β_c_ (V dec^−1^)	Corrosion rate (m/y)	Corrosion Resist. (Ω)
As-received	0.285	317.4	75	125	0.261	71.42
Uncoated	0.275	440	120	185	0.252	114.93
Heated at 35 °C	0.225	450.1	112	230	0.207	145.36
Heated at 100 °C	0.220	510.2	150	290	0.201	195.12
Heated at 200 °C	0.255	498.6	165	312	0.233	183.8
Heated at 300 °C	0.270	448	140	187	0.247	125.8

From Fig. 5 and Table 2, it can be observed that the sample heated to 100 °C exhibited the highest potential shift in the positive direction, followed by the sample which was heated at 35 °C. According to^4^, this means that the sample heated to 100 °C and the sample heated to 35 °C showed more corrosion resistance than other samples. This is further confirmed by the highest corrosion resistance values and lowest corrosion rates of 195.12 Ω, 0.201 m/y and 145.36 Ω, 0.207 m/y respectively displayed by samples heated to 100 °C and the sample heated to 35 °C. This is in-line with the work of^10^.

However, the sample heated to 200 °C showed burning effect which resulted to crack initiation on the coating surface. Hence, it was obvious that chloride ions and other corrosion causing agents in the corrosion medium have passed through to the mild steel surface thus responsible for the decrease in corrosion resistance and increased corrosion rate of the sample. At 300 °C, the coating on the surface of the sample was completely decomposed.

Generally, the rise in temperature reduced the particle agglomeration which in turn reduced the cracks and voids tendencies of nanoparticle coating and consequently improving the adhesion at substrate/coating interface ultimately reducing corrosion rate.

### EDS analysis of the steel samples

The elemental composition obtained from EDS analysis of all the samples under investigation before and after corrosion is shown in Figs. 7, 9, 11, 13, 15 and 17 while Figs. 6, 8, 10, 12, 14 and 16 shows the SEM cross sectional view.

#### As-received sample

**Figure 6:**
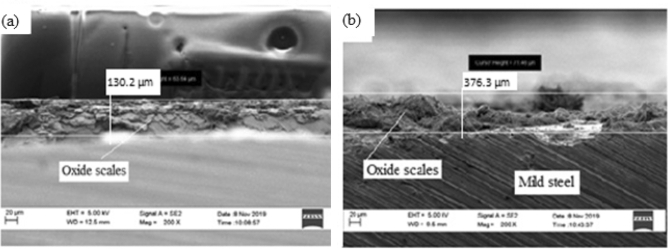
Microstructure of the cross-sectional view of as-received mild steel sample (**a**) before corrosion test (**b**) after corrosion.

Figure 6 shows high resolution scanning electron microscopy cross-section images of as-received sample before and after corrosion.

**Figure 7:**
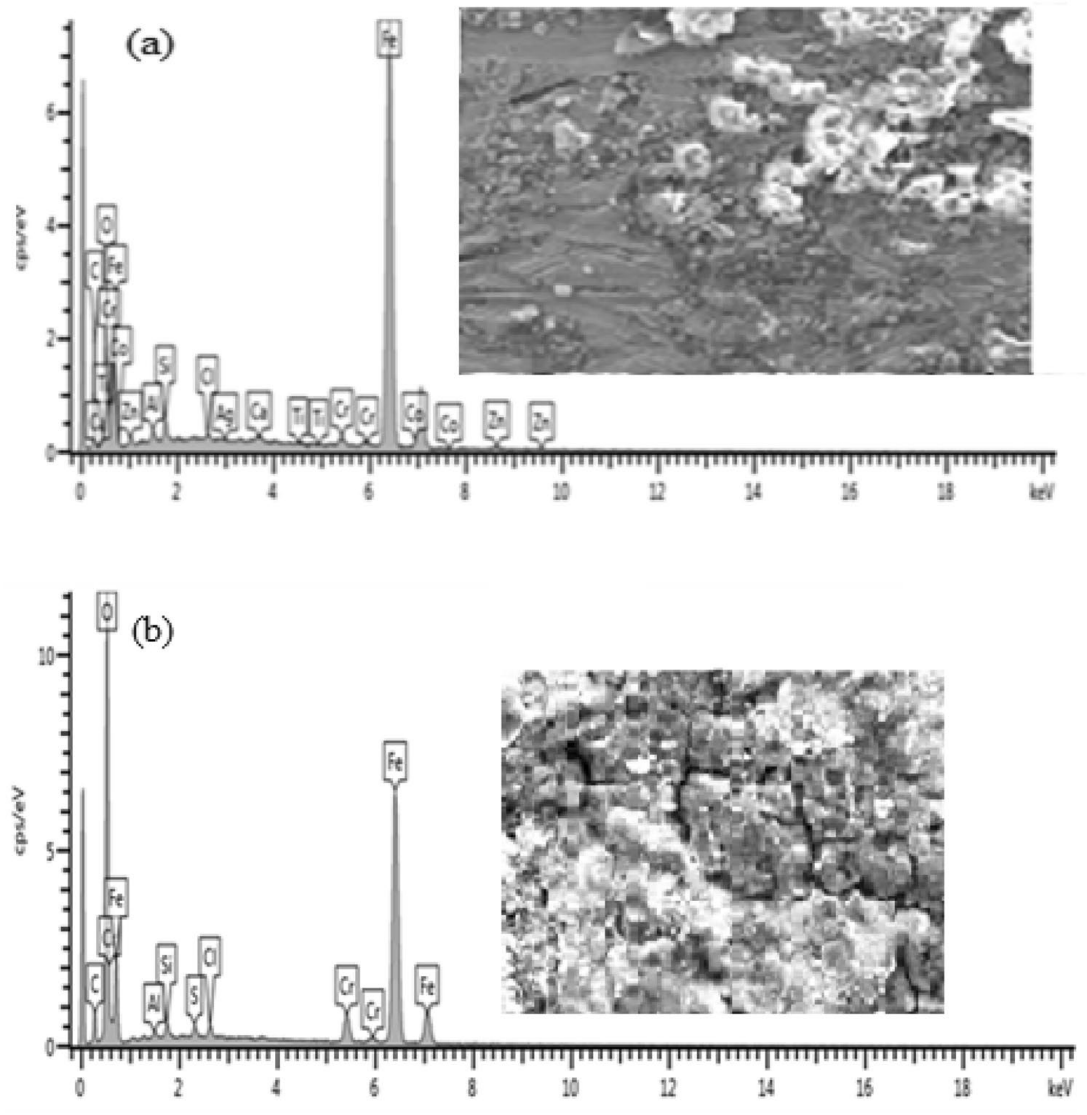
Energy diffraction spectron (EDS) of As-received sample: (**a**) before corrosion and (**b**) after corrosion.

The corrosion scales observed on Fig. 6(a) was due to interaction of mild steel with the environment which resulted to the oxidation of the surface. The image also revealed the existence of single layer of 130.2 µm thick, compact and crystalline rust products. After the sample was subjected to corrosion test, an increased layer of dense and non-uniform corrosion scales of 376.3 µm thickness was noticed. This shows that there is increased corrosion rate as a result of oxidation reaction from sea water constituents. The elemental composition of the rust layers obtained from EDS analysis of as-received samples before and after corrosion is shown in Fig. 7(a),(b).

From Fig. 7, the presence of Fe and O is an indication of iron oxides formation in the form of Fe_2_O_3_ and Fe_3_O_4_. It can be observed from Fig. 7 that there was decrease in the percentage composition of iron from 59.51 wt% before corrosion to 51.13 wt% after corrosion and increase in percentage composition of oxygen from 14.42 to 32.95 wt% respectively. This shows that more iron has been oxidised to form a thicker layer of hydroxide which accounted for severity of corrosion in the sample.

#### Uncoated heat-treated sample

**Figure 8:**
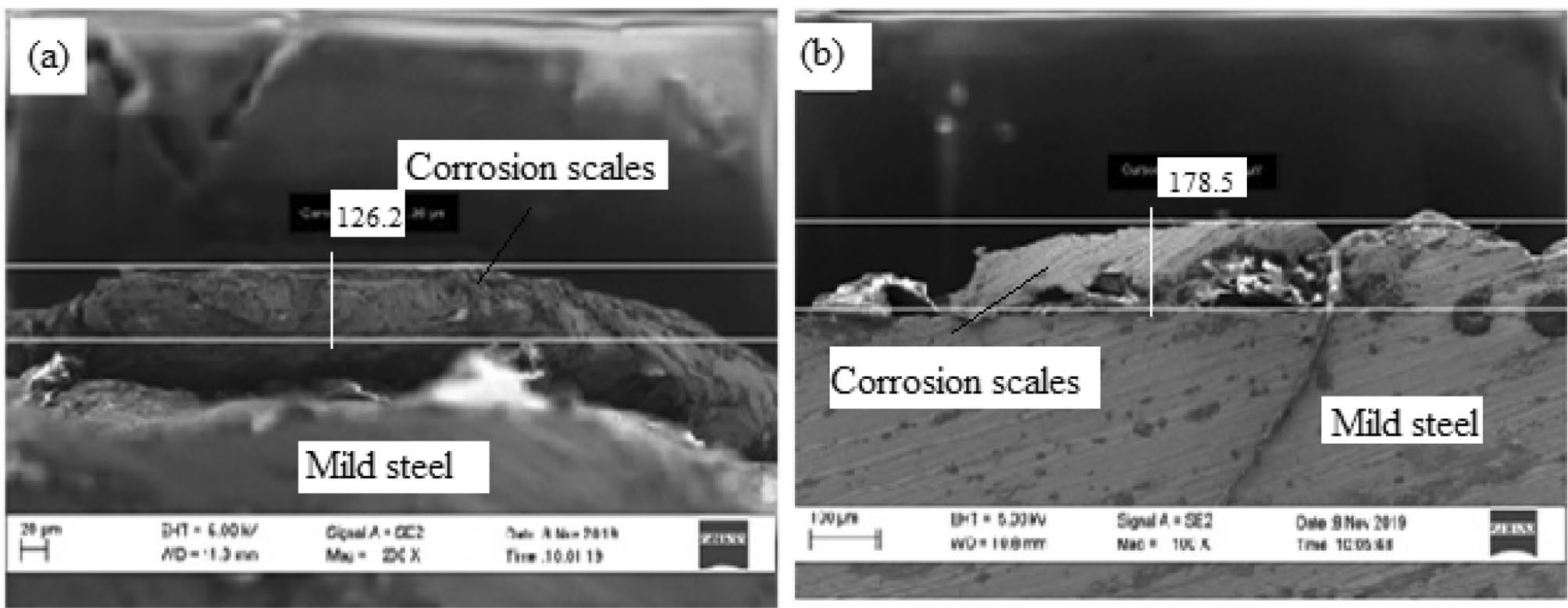
SEM surface view of annealed sample at 850 °C (**a**) before and (**b**) after corrosion.

Figure 8 shows high resolution scanning electron microscope images of heat-treated sample at 850 °C before and after corrosion.

**Figure 9:**
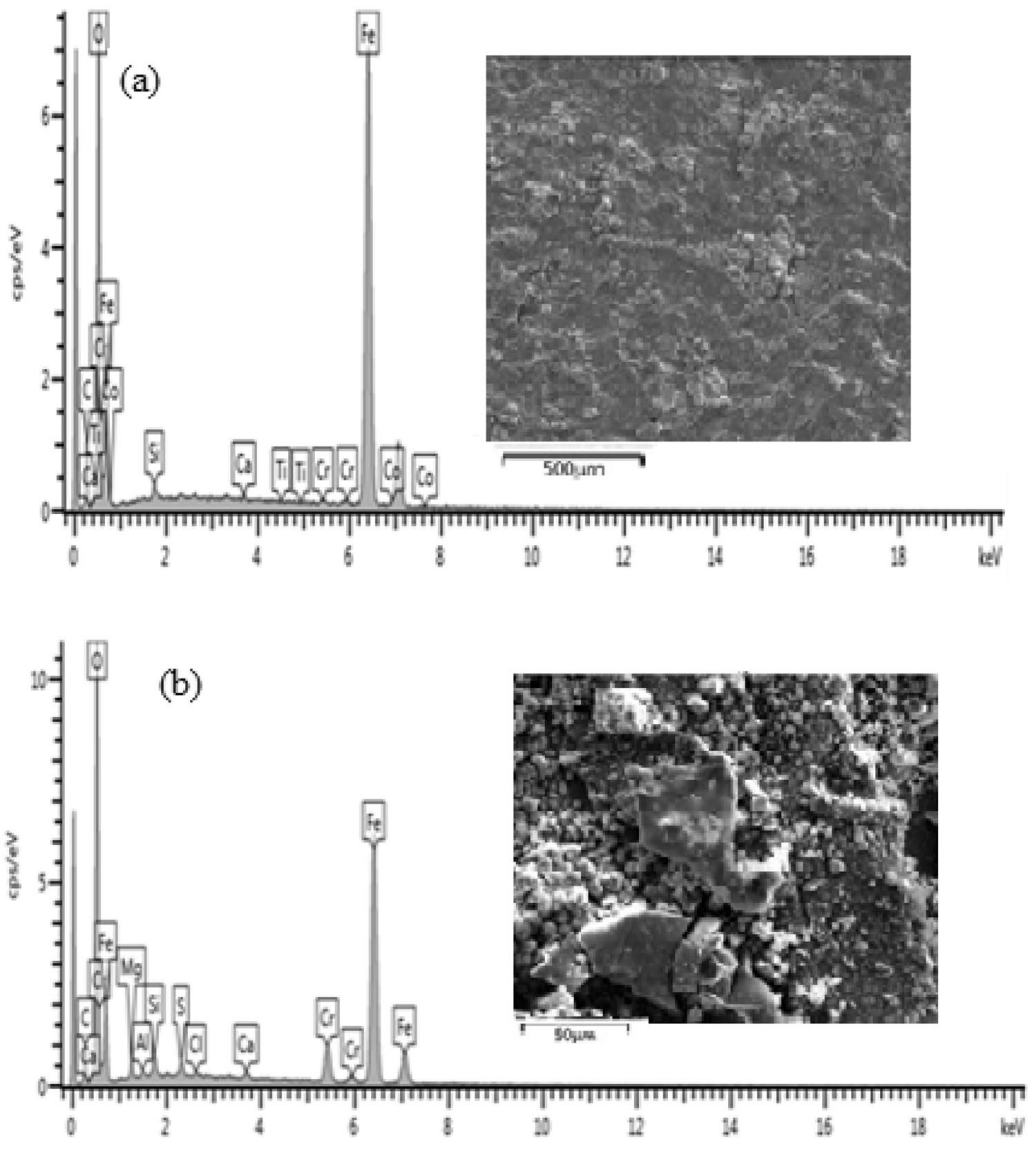
Energy diffraction spectron (EDS) of annealed sample: (**a**) before corrosion and (**b**) after corrosion.

Figure 8(a) shows a layer of corrosion scales of 126 µm thick while the SEM view in Fig. 8(b) shows a thicker corrosion scale of 178.5 µm thick. It can be observed that corrosion intensity is not as severe as compared to that of as-received sample. The reduction in the formation of oxide layer was due to increased resistance of the specimen occasioned by micro structural modification from heat treatment^12^. Barchiche et al.^13^ reported similar reduction of corrosion rate of mid steel in apple juice due to surface hardening as a result of heat treatment. The elemental composition of the rust layers obtained from EDS analysis of the samples before and after corrosion is shown in Fig. 9(a),(b).

From Fig. 9, it can be observed that there is only 3.31w% percentage reduction in the Fe content between the samples before corrosion and after corrosion as compared to 8.38 percent for as-received sample. This is an indication that corrosion is suppressed as a result of heat treatment of the sample.

#### Coated sample and heated at 35 °C

**Figure 10:**
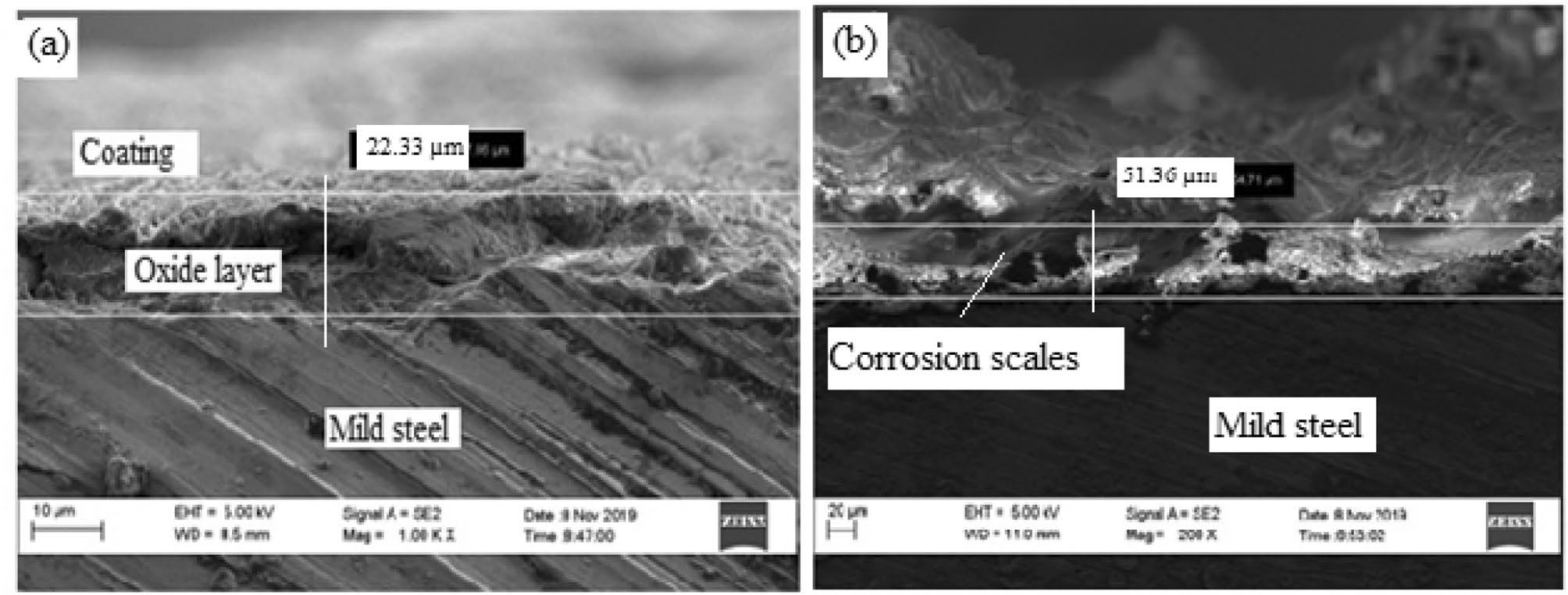
SEM surface view of sample annealed at 850 °C, coated and cured at 35 °C: (**a**) before corrosion, (**b**) after corrosion.

Figure 10 shows high resolution scanning electron microscope images of the sample heated at 35 °C before and after corrosion.

**Figure 11:**
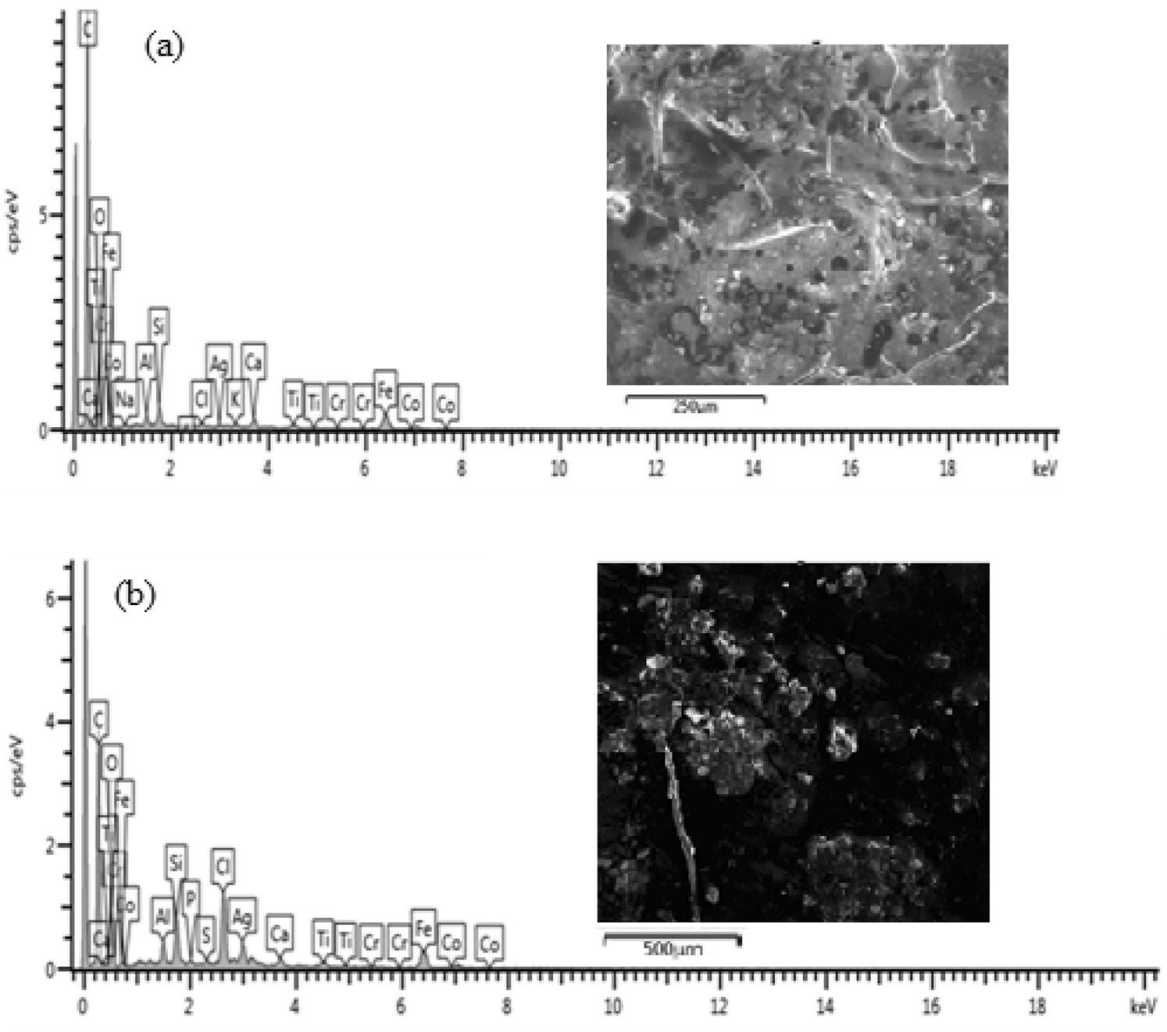
Energy diffraction spectron (EDS) of sample cured at 35 °C: (**a**) before corrosion and (b) after corrosion.

From Fig. 10(a) it can be observed that the surface was covered with spongy – like coating membrane that remains loosely adhere to the surface. It can be understood that the room temperature curing has not provided adequate adherence at the coating/substrate interface. A corrosion scale of 22.33 µm thickness was noticed before the sample was subjected to corrosion test. Figure 10(b) shows an increased thickness of corrosion scales of 51.36 µm. This means that there is permeation of corrosion medium across the coating surface. The reaction of corrosion medium with the coating resulted to formation of voids and micro cracks which created access of corrosion medium to the substrate therefore causing dissolution of metal and forming oxides^14^. It can be observed that the corrosion attack is not as severe as seen in the former two cases thus the coating can be seen to be responsible for the reduced corrosion attack on the specimen. The elemental composition of the rust layers obtained from EDS analysis of the samples before and after corrosion is shown in Fig. 11(a),(b).

From Fig. 11, comparing the values of Fe and O before and after corrosion, it can be observed that there is reduction in percentage weight of Fe from 55.65 to 49.55 and increased percentage weight of O from 16.22 to 19.46. This signifies that there is oxidation of Fe due to corrosion^15^. The percentage differences of 6.1 wt% for Fe and 3.24 wt% for O is an indication that there is restriction to the formation of oxide scales due to existing barrier between the corrosion medium and the substrate.

#### Coated sample and heated at 100 °C

**Figure 12:**
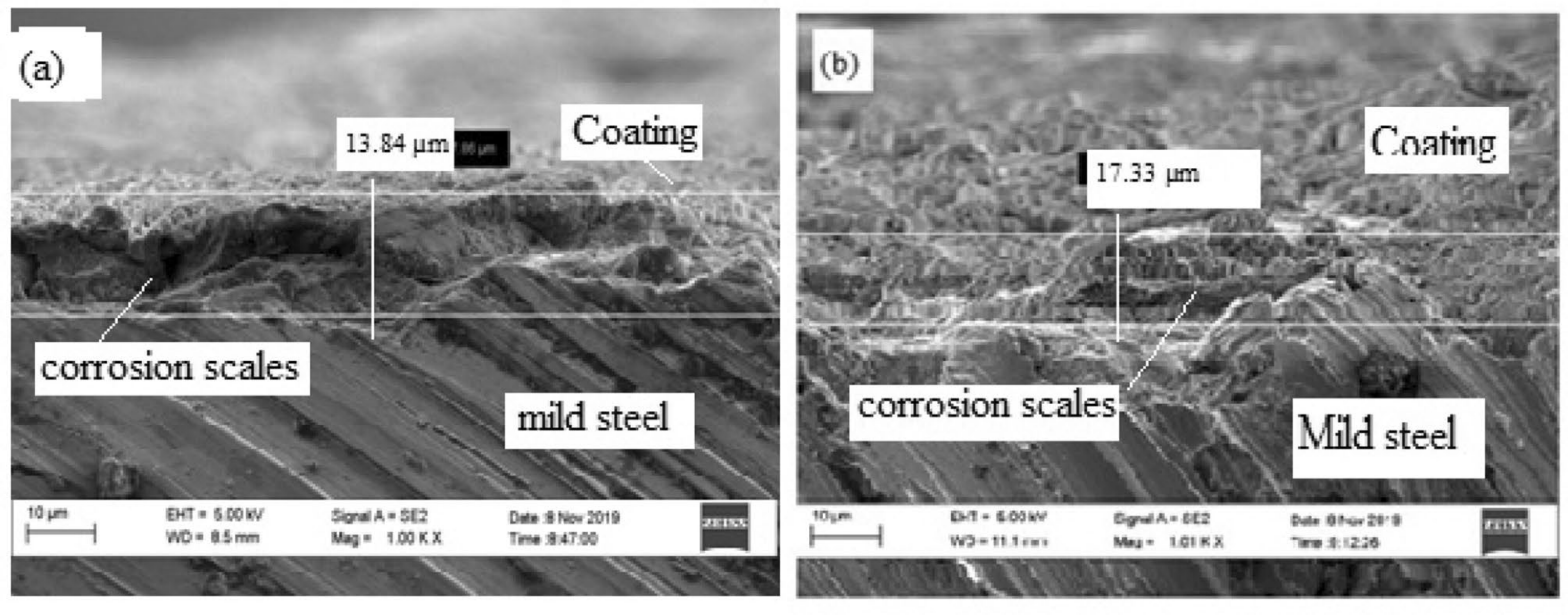
SEM cross-sectional view of the sample coated and heated at 100 °C (**a**) before corrosion and (**b**) after corrosion.

Figure 12 shows the HRSEM micrographs of the sample treated at 100 °C.

**Figure 13:**
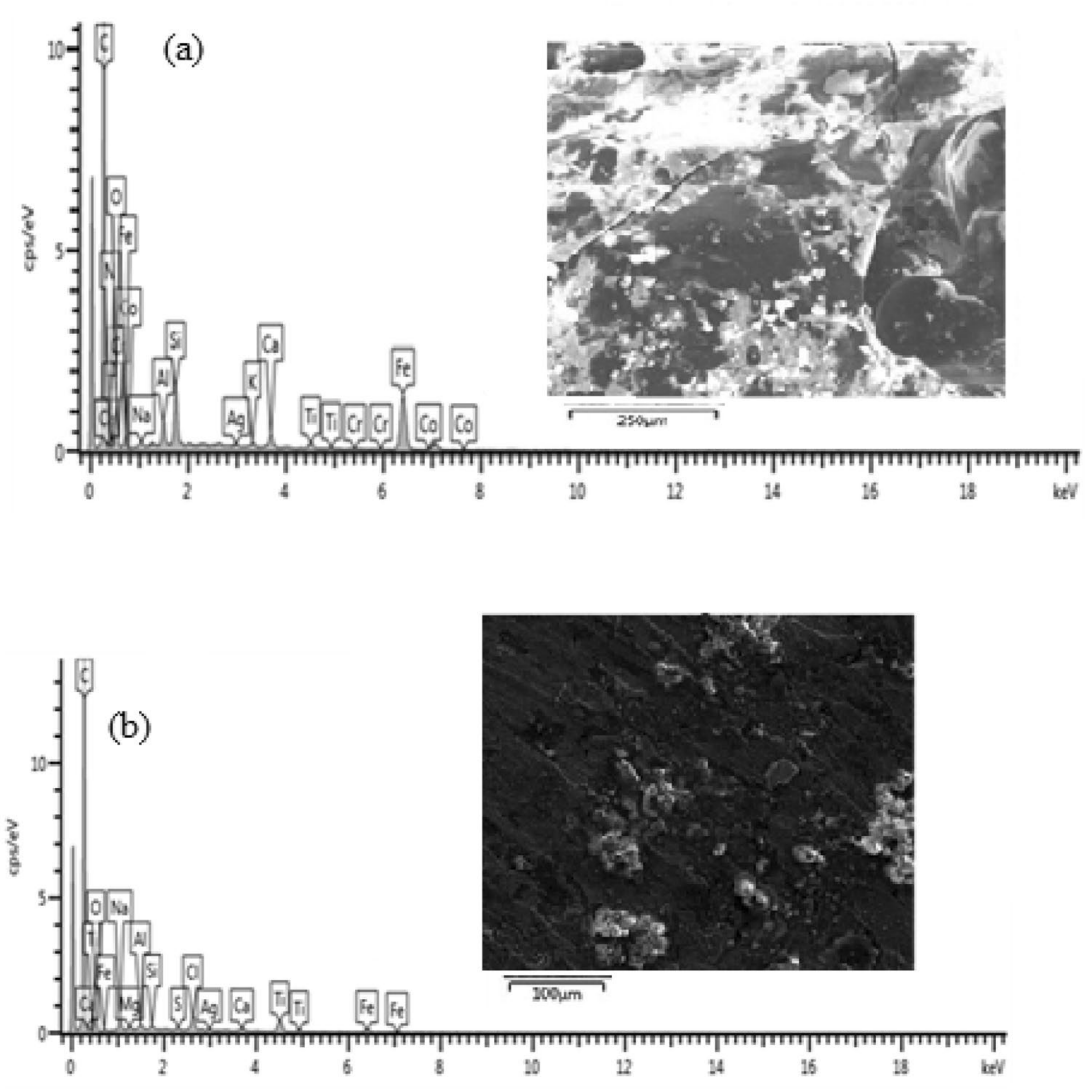
Energy diffraction spectron (EDS) of sample cured at 100 °C: (**a**) before corrosion and (**b**) after corrosion.

From Fig. 12(a) at 100 °C a smooth layer of top coat with the corroded layer of 13.84 µm thickness can be observed. The application of heat at 100 °C resulted in simultaneous promotion of cross-linkage of the coating network over the base material thus providing stronger adhesion and formation of tough coat over the substrate which effectively suppressed the corrosion causing agents. This was evident by the corrosion layer of 17.33 µm obtained after the specimen was subjected to potentio-dynamic polarization corrosion test. The result obtained from the SEM images shown in Fig. 10 is in agreement with the potentio-dynamic polarization tafel plot which shows that the sample coated and heated at 100 °C has the least negative potential hence, less susceptible to corrosion. It can be observed that the coating on the sample heated at 100 °C had better contact with base material and this is attributed to higher plastic deformation that resulted from the temperature treatment^12^. The elemental composition of the corrosion scales obtained from EDS analysis of the samples before and after corrosion is shown in Fig. 13(a),(b).

From Fig. 13(a),(b), it can be observed that the percentage weight difference of Fe and O before and after corrosion is 1wt% and 0.84wt% respectively. The slight difference is an indication that the amount of rust product formed is very minimal as compared to other samples. Thus, the corrosion of the specimen in the medium was highly suppressed by the coating membrane which is also an indication of stronger adhesion of the coating to the substrate^9^.

#### Coated sample and heated at 200 °C

**Figure 14:**
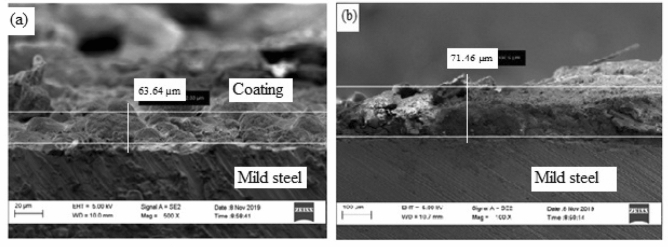
SEM cross-sectional view of the sample coated and heated at 200 °C (**a**) before corrosion and (**b**) after corrosion.

Figure 14 shows the HRSEM micrographs of the sample treated at 200 °C is shown in.

**Figure 15:**
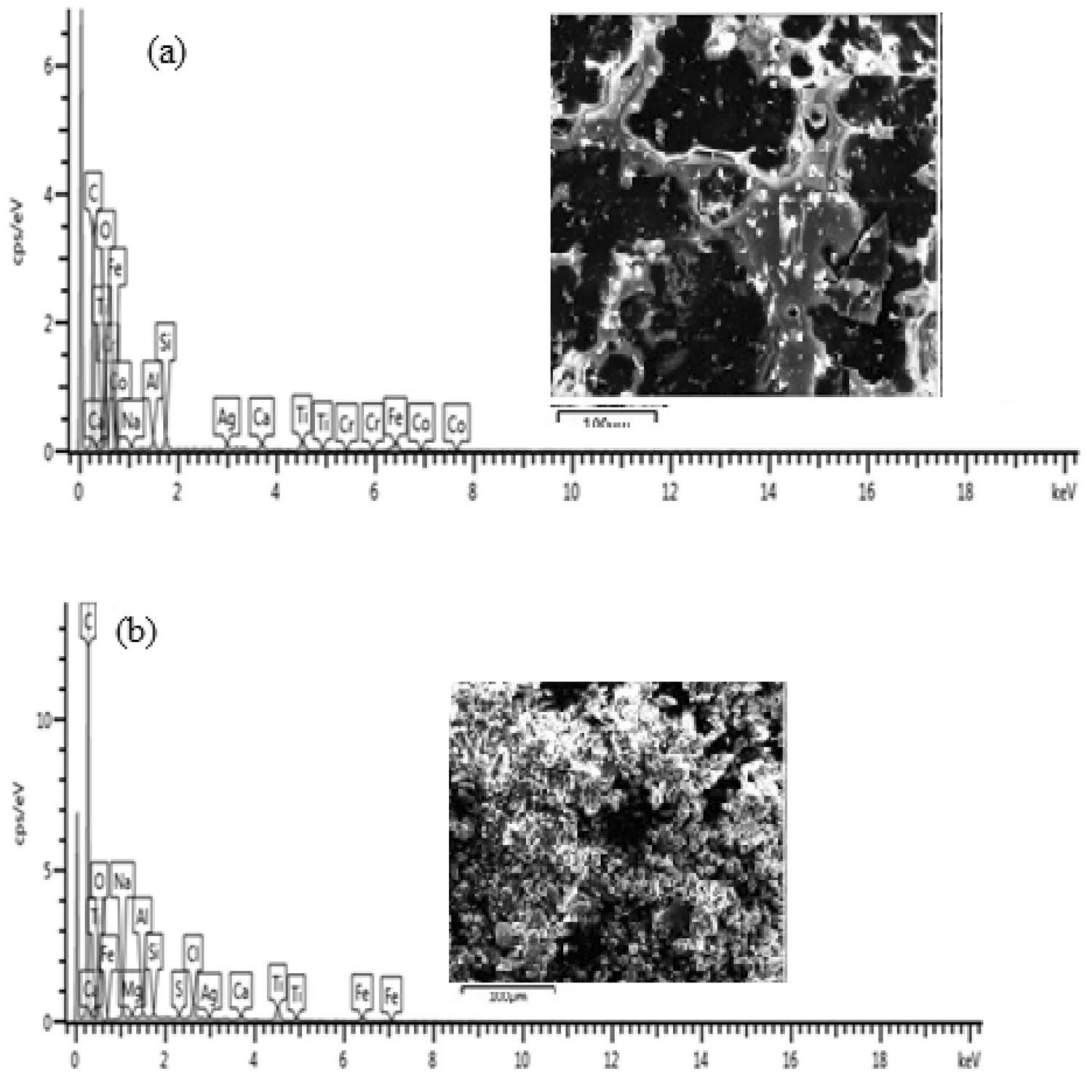
Energy diffraction spectron (EDS) of sample cured at 200 °C: (**a**) before corrosion and (**b**) after corrosion.

From Fig. 14(a) at 200 °C the coating on the surface of the sample became damaged due to the burning of the epoxy resin used as the binder. It was observed that the thickness of the corroded layer is 63.64 µm. According to literature, the glass transition temperature of epoxy resin (165 °C) was exceeded thus, causing the depletion of the coating. As a consequence of coating depletion, various sizes of pores were created which served as active sites for corrosion initiation and propagation. After the specimen was subjected to corrosion medium, massive corrosion scales were observed to a value of 71.46 µm as shown in Fig. 14(b). The corrosion intensity depicted in the image shows good agreement with the Tafel plots which indicated that the sample treated at 200 °C is more susceptibility to corrosion than the samples treated at 35 °C and 100 °C respectively. The elemental composition of the corrosion scales obtained from EDS analysis of the samples before and after corrosion is shown in Fig. 15(a),(b).

From the EDS analysis of the sample, it can be observed that the percentage composition of Fe has been reduced by 4.38 wt% in the presence of 27.15 wt% O. This is an indication that the rate of corrosion is relatively high. The high rate of corrosion is caused by the thermal degradation of the coating at 200 °C.

#### Sample heated to 850 °C, coated and cured at 300 °C

**Figure 16:**
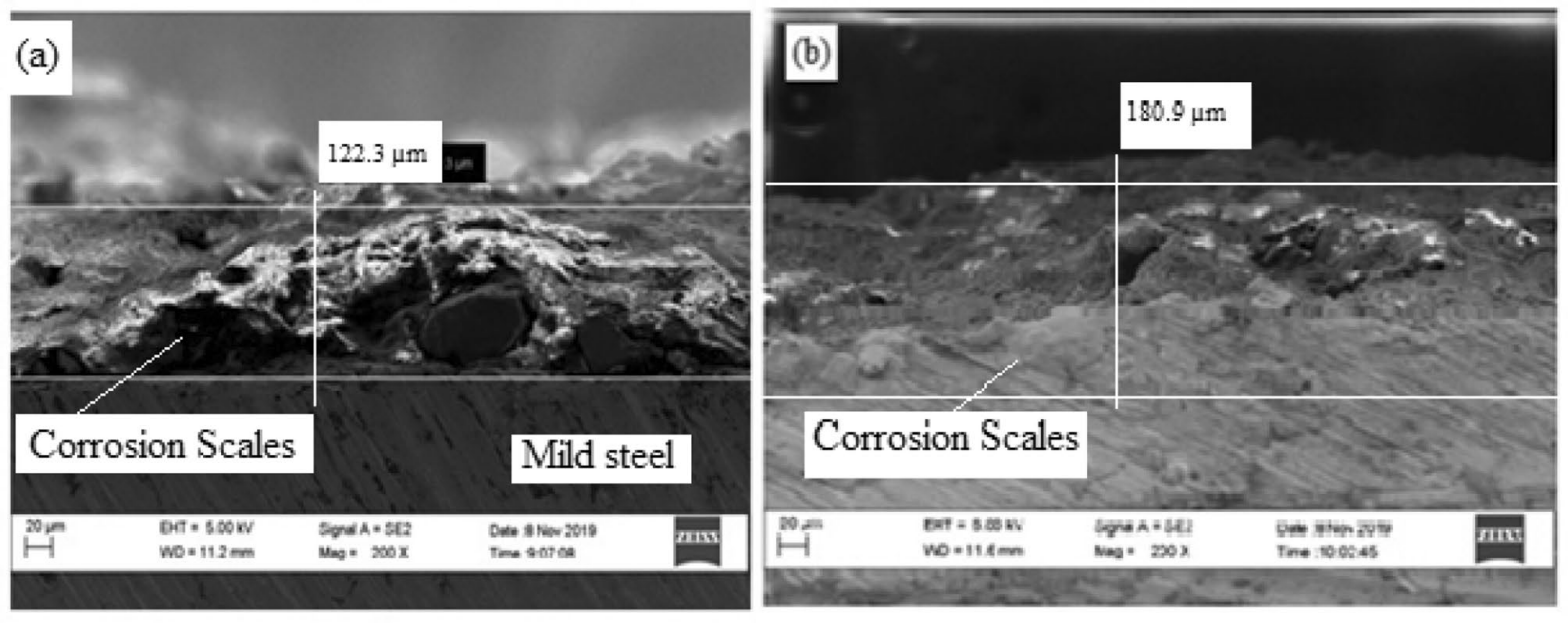
SEM cross-sectional view of the sample coated and heated at 300 °C (**a**) before corrosion and (**b**) after corrosion.

Figure 16 shows High resolution scanning electron microscope images of the sample treated at 300 °C before and after corrosion.

**Figure 17:**
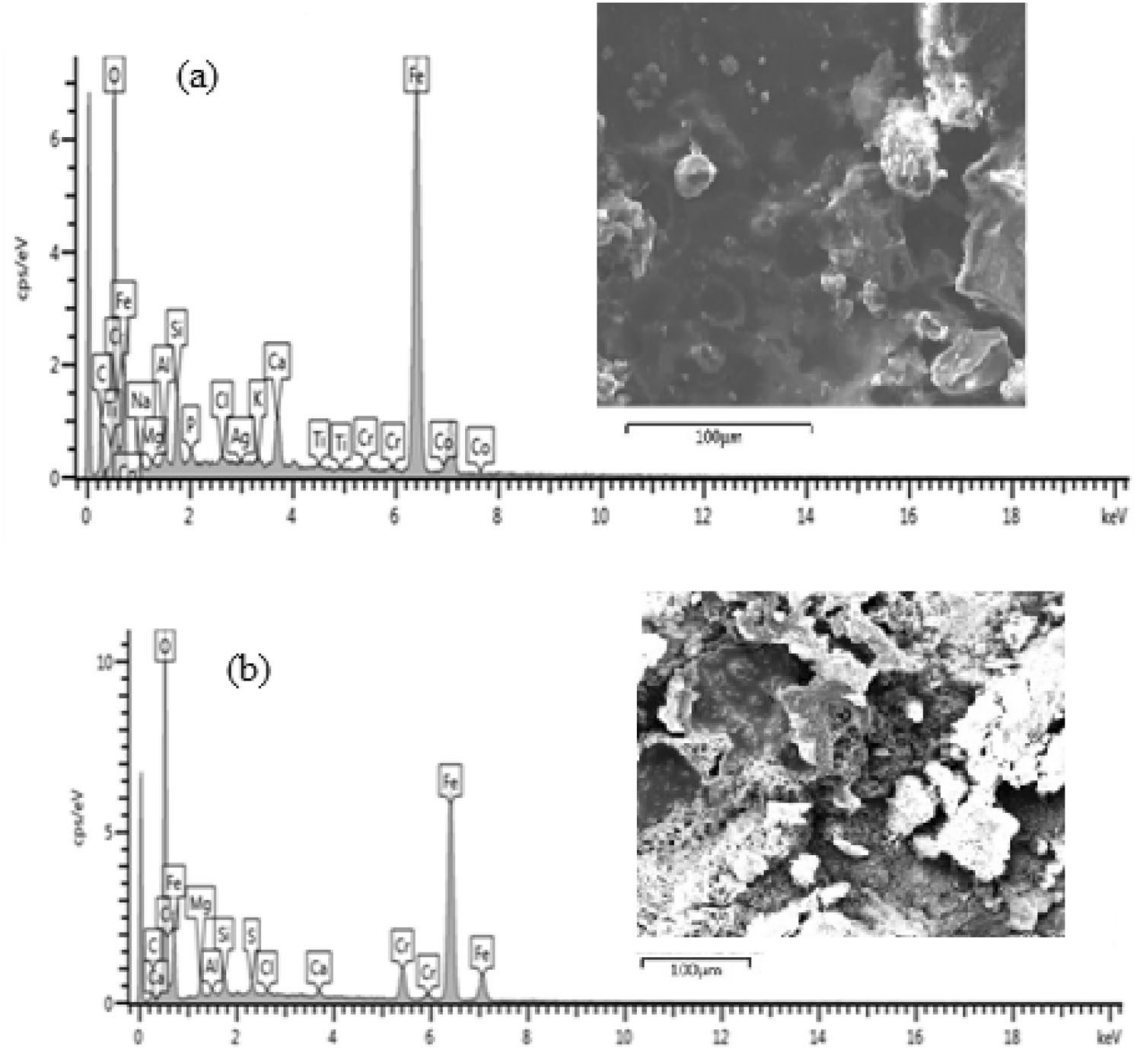
Energy diffraction spectron (EDS) of sample heated at 200 °C: (**a**) before corrosion and (**b**) after corrosion.

From Fig. 16(a), at 300 °C, a thick, dense corrosion layer of 122.3 µm was observed. The coating was completely delaminated, thereby exposing the metal to severe corrosion. After the potentio-dynamic polarization test the thickness of the oxide scales increased to 180.9 µm as shown in Fig. 16(b). This shows that the sample was highly susceptibility to corrosion. From Table 2, there is a similarity between the current potentials of samples heated at 300 °C and the as-received samples which can be attributed to the degradation of the coating from the surface of the specimen. The elemental composition of the corrosion scales obtained from EDS analysis of the samples before and after corrosion is shown in Fig. 17(a),(b).

## Conclusion

In this study, Silver–Cobalt Oxide–Titanium Dioxode (Ag/Co_3_O_4_/TiO_2_) nanocomposites was synthesized, characterized and coated on AISI 1020 in order to evaluate the corrosion resistance under high temperature condition. On the basis of the results of the investigation, the following conclusions are drawn:

1. Excellent reduction in particle agglomeration and improvement in coating adhesion at substrate/coating interface was achieved at temperatures of 35 °C and 100 °C.
2. Samples treated at 100 °C exhibited highest potential shift in the positive direction and showed losser corrosion rate of 0.201 m/y and highest corrosion resistance of 195.12 Ω.
3. The optimum corrosion resistance of (Ag/Co_3_O_4_/TiO_2_) nanocomposite coating on AISI 1020 was achieved at temperature of 100 °C.
